# Mind–body exercise for symptom management in cancer: a systematic review and meta-analysis

**DOI:** 10.3389/fpubh.2026.1762140

**Published:** 2026-03-20

**Authors:** Xinmei Liu, Hongfan Qiu, Yuxuan Tao, Xiaoyu Jiang, Bingjie Wang, Chenglong Yao, Haixia Li

**Affiliations:** 1 ShanDong University of Traditional Chinese Medicine, Jinan, China; 2 China Academy of Traditional Chinese Medicine Guang’anmen Hospital, Beijing, China; 3 Beijing Jishuitan Hospital, Capital Medical University, Beijing, China

**Keywords:** cancer, health-related quality of life, mental health, meta-analysis, mind–body exercise, physical symptoms

## Abstract

**Objective:**

The aim of this study is to conduct a systematic review and meta-analysis and to evaluate changes in the mental health, physical symptoms, and health-related quality of life (HRQOL) in cancer patients following mind–body exercise training.

**Methods:**

As of February 2026, this study retrieved data from eight databases. Eventually, 19 randomized clinical trials using the mind–body exercise were conducted. This meta-analysis synthesized outcome indicators related to cancer symptoms to obtain pooled effect sizes.

**Results:**

In this meta-analysis, interventions in mind–body exercise in terms of anxiety (standardized mean difference, SMD = −0.39, 95% CI −0.65 to −0.13), depression (SMD = −0.34, 95%CI −0.59 to −0.10), fatigue (SMD = −0.40, 95% CI −0.78 to −0.02), sleep (SMD = −0.60, 95% CI −1.15 to −0.05), and HRQOL (SMD = 0.38, 95% CI 0.17–0.60) are statistically significant when compared with usual care. The subgroup analysis indicated that a longer intervention period (≥ 8 weeks) might show stronger effects. Based on the intervention types, traditional Chinese exercise significantly improved anxiety, depression, sleep quality, and HRQOL, while yoga significantly reduced fatigue and also improved HRQOL.

**Conclusion:**

Mind–body exercise is an effective rehabilitation method for alleviating cancer symptoms, significantly improving mental health, physical symptoms and quality of life. They are suitable as non-pharmacological supplementary intervention measures for cancer patients and are recommended for wide application in clinical practice.

## 1 Introduction

Nowadays, with improvements in cancer treatment technology, the number of cancer survivors is increasing rapidly. However, the recovery phase is frequently associated with enduring psychological and physical aftereffects. Epidemiological studies have shown that a significant number of cancer survivors report experiencing psychological, physical, and daily life-related problems (1–3).

At present, traditional drugs or psychotherapy still have limitations (side effects, access, and cost). A recent review of anxiety and depression in cancer convalescents points out that the evidence of drug treatment is inconsistent (4). In addition, many tumor drug trials have failed to demonstrate concurrent improvements in patients’ health-related quality of life (HRQOL) (5). Similarly, a clinical review of insomnia in cancer patients emphasizes that although hypnotic drugs may provide short-term relief, evidence of their long-term benefits and safety in these patients remains limited (6). Therefore, it is essential to seek an intervention measure that is both safe, scalable, and multi-dimensional. Engaging in physical activity after diagnosis can help alleviate anxiety, depression, fatigue, improve sleep, and enhance HRQOL (7). In addition, exercise is safe both during and after cancer treatment, and survivors are recommended to “avoid inactivity” (8).

In this case, mind–body exercise—training that integrates bodily movement, mindful breathing, and meditative awareness—provides a promising non-pharmaceutical strategy for cancer rehabilitation. Mind–body exercise like yoga, Qigong, and Tai Chi blend gentle movements with mindfulness techniques, offering a one-two punch against both mental health challenges (like anxiety and depression) and physical complaints (such as fatigue and poor sleep quality) (9–11). Prior evidence suggests that the effects of mind–body exercise may vary by dose and duration, intervention type, outcome selection and measurement, and comparator choice, contributing to heterogeneity across trials. Reviews from non-oncology populations provide useful methodological benchmarks, highlighting the importance of intervention dose, delivery, and adherence, and the challenges of subjective, scale-based outcomes. Trials using standardized intervention delivery and validated stress-related endpoints further illustrate how clearer dose reporting improves interpretability. In oncology, existing syntheses suggest potential benefits of yoga and traditional Chinese exercise for symptom management, but evidence remains fragmented across outcomes and intervention formats, with variable certainty. Therefore, a comprehensive synthesis with transparent risk-of-bias and certainty appraisal is warranted. This review focused on patients with cancer, compared mind–body exercise with usual care, evaluated a symptom-cluster set of outcomes, and explored effects depending on intervention type and duration. Detailed information is provided in Table 1.

**Table 1:** Comparison with prior reviews and evidence.

Prior evidence	Theme	Key implication for the present review
Zou et al. (50), Zhang et al. (51), and Zou et al. (52)	Dose/duration may influence detectable effects	Motivated duration subgroup (< 8 vs. ≥ 8 weeks) and cautious interpretation of short interventions
Zou et al. (52) and Hou et al. (53)	Intervention type and delivery/adherence may contribute to heterogeneity	Supported subgrouping by intervention type and consideration of adherence/delivery as potential heterogeneity sources
Zou et al. (54)	Selection of the control group affects estimated effects and certainty	Comparator restricted to usual care and certainty appraised with GRADE
Zou et al. (55)	Outcome domains and measurement tools shape conclusions	Supported a symptom-cluster framework and standardized synthesis using SMD
Yue et al. (56)	Mechanistic plausibility through stress regulation/neuroplasticity	Provided mechanistic rationale for psychological and sleep-related outcomes
Zhu et al. (57)	Standardized dose delivery and validated endpoints can strengthen interpretability	Reinforced the value of reporting intervention dose (frequency/session length/weeks) and using validated scales
Huang et al. (58)	Common methodological limitations across populations	Justified ROB2 assessment and multi-strategy sensitivity analyses for subjective outcomes
Kuo et al. (59), Hou et al. (53), Liu et al. (60), and Niu et al. (61)	Oncology evidence suggests potential benefits but remains heterogeneous	Supported comprehensive cancer-focused synthesis spanning multiple outcomes and intervention types

SMD, Standardized mean difference; ROB2, Risk of bias 2.0; GRADE, Grading of Recommendations Assessment, Development and Evaluation.

## 2 Methods

The meta-analysis was conducted based on existing randomized clinical trials (RCTs). Therefore, the institutional review board approval and individual informed consent form were not required.

### 2.1 PROSPERO registration

This article was registered with the International Prospective Register of Systematic Reviews (PROSPERO; under registration number 420251106396). This meta-analysis was conducted according to the guidelines outlined in the Preferred Reporting Items for Systematic Reviews and Meta-Analyses (PRISMA) (12) and the Cochrane Handbook (13).

### 2.2 Literature retrieval strategy

Literature searches were conducted in eight electronic databases, including PubMed, Embase, the Cochrane Library, Psychological Information (PsycINFO), Web of Science, China National Knowledge Infrastructure (CNKI), Wanfang Data, and Chinese Scientific Journals Database (VIP). The initial search was conducted from database inception to July 2025. During revision, the search was updated, and the final search was completed on 25 February, 2026. The search used keywords related to mind–body exercise, including “Mind Body,” “Traditional Chinese Exercise,” “Yoga,” “Tai Chi,” “Qigong,” “Baduanjin,” “Yijinjing,” and “Wuqinxi.” Cancer-related terms included “Neoplasms,” “Tumors,” and “Cancer.” Outcome terms focused on “Anxiety,” “Depression,” and “Quality of Life.” The full electronic search strategies for all databases are provided in Supplementary Table 1.

### 2.3 Study criteria and screening process

At excluding duplicates documents using EndNote X9 software, screening started with title and abstract review, followed by full-text evaluation against predetermined inclusion and exclusion parameters. The specific process is illustrated in Supplementary Figure 1. Inclusion criteria included patient/population, intervention, comparator, outcome, and study design (PICOS). The population included patients with cancer. The intervention measures were defined as various forms of mind–body exercise, including yoga, Tai Chi, Baduanjin, and Qigong, while the control group received usual care. The primary outcomes included anxiety, depression, and/or HRQOL, with no secondary outcomes. Qualified studies included RCTs or parallel-design RCTs. Additional requirements included full-text availability and peer-review status. Exclusion criteria comprised animal-based researches, theoretical studies, reviews, conference abstracts, or research reports. Non-randomized controlled trials (NRCT) were also excluded. If a study included multiple interventions, only the comparison between mind–body exercise and usual care was considered.

### 2.4 Data extraction and integration

Two authors (X. L. and H. Q.) independently conducted data extraction. Discrepancies were resolved by discussion with a third author (B. W.). The compiled research data contained the researcher’s name, publication year, methodology, and participant group count. Research characteristics include sample size, age, scale, and score used for outcome indicators. The characteristics of the intervention include the intervention method and duration. In the final phase of data acquisition, all quantitative outcome measures were systematically extracted.

The following summary statistics were extracted for post-intervention: sample sizes, means, and standard deviations (SD). When continuous outcomes were not directly reported as means with SDs, appropriate statistical conversions were applied. For studies reporting standard errors (SEs), SDs were calculated using the formula SD = SE × √n. For studies presenting 95% confidence intervals (95% CI), SDs were derived from the standard errors calculated from the CIs according to the recommendations of the Cochrane Handbook (13). When outcomes were reported as medians with interquartile ranges (IQRs), ranges, or other dispersion measures, both means and SDs were estimated using methods proposed by Wan et al. (14) and Luo et al. (15). These approaches provide validated formulas for approximating means and SDs from medians, ranges, and IQRs under the assumption of approximately symmetric distributions. All conversions were conducted to standardize outcome data across studies for meta-analysis. Sensitivity analyses excluding studies with imputed SDs were performed to assess the robustness of the pooled estimates.

This study also extracted the post-intervention values (i.e., the outcome indicators at the end of the intervention period) for a meta-analysis. In all the included trials, the baseline characteristics and baseline outcome indicators of the intervention group and the control group were reported as being comparable (*p* > 0.05). Therefore, the comparison between the groups after the intervention is considered applicable for estimating the treatment effect.

For multi-arm trials, only the intervention arms that met the predefined inclusion criteria were extracted. When other arms did not meet the eligibility criteria, only the eligible intervention arm and the shared control group were included. Because only one eligible comparison from each study was incorporated into the meta-analysis, no adjustment of the control group sample size was required. This approach is consistent with the recommendations of the Cochrane Handbook. No study contributed more than one comparison to the same pooled analysis.

### 2.5 Outcome and definition of mind–body exercise and quality assessment

This study defines “mind–body exercise” as a structured training approach, which includes the following three elements, including (1) gentle-to-moderate physical movement, (2) regular breathing control, and (3) a component of concentration or meditation consciousness. Therefore, movement disciplines such as yoga, Tai Chi, Qigong, and Baduanjin were encompassed within this category. This study explicitly excluded those studies based on meditation-based or mindfulness-based stress reduction (MBSR) programs without an exercise component, because these represent mind–body therapies but not mind–body exercise. This decision was to improve conceptual clarity and reduce methodological heterogeneity, so that the comprehensive analysis focuses on interventions that include substantial physical activity elements in addition to concentration.

This study included RCTs, the Cochrane Risk of Bias Tool 2.0 (ROB 2.0) to assess the biases present in the selected studies. Two researchers working—X. L. and H. Q.—independently conducted this assessment. Any inconsistencies between them were resolved by discussion with the third member, B. W.

### 2.6 Statistical analysis

RevMan5.4 software was used to analyze the 19 literature data and generate forest plots. The data were analyzed by the random-effects meta-analysis model. Effect values were presented as standardized mean difference (SMD) and 95% CI. Statistical analysis was conducted using a two-tailed test and *p* < 0.05 is considered statistically significant. Forest plots were drawn to visually present the meta-analysis results. Heterogeneity across studies was assessed using the I^2^ statistic; values ≤75% indicate low to moderate heterogeneity, while values >75% indicate high heterogeneity.

### 2.7 Subgroup analysis

Subgroup analyses were conducted by intervention type and intervention duration, based on clinical relevance and previous literature suggesting potential dose–response effects. These subgroup analyses were exploratory and not pre-specified in the PROSPERO registration, and findings were interpreted cautiously.

### 2.8 Publication bias

Regarding publication bias, since the Egger test has relatively low test efficacy when the number of studies is less than 10, this study conducted a visual inspection of the funnel plot for the results of at least 10 studies. For the results of studies with fewer than 10 studies, only visual assessment was used, and it was impossible to reliably evaluate publication bias.

### 2.9 Sensitivity/influence analysis

Sensitivity analysis were performed to assess the robustness of the pooled estimates. Firstly, a leave-one-out approach was applied by sequentially removing one study at a time and re-estimating the pooled effect. Secondly, analyses were repeated after excluding studies rated as high risk of bias according to ROB 2.0. Thirdly, analyses were repeated after excluding studies in which SDs were derived/estimated from other summary statistics. The direction and statistical significance of pooled effects were compared across sensitivity analyses to evaluate the stability of the findings.

### 2.10 Certainty of evidence

The certainty of evidence for each outcome was assessed using the GRADE approach (16). Evidence from randomized controlled trials was initially rated as high certainty and downgraded, when appropriate, for risk of bias, inconsistency, indirectness, imprecision, and publication bias. Overall certainty was rated as high, moderate, low, or very low and summarized in a GRADE evidence profile.

## 3 Results

### 3.1 Literature selection

As of 25 February, 2026, a total of 7,098 articles were identified in the initial screening. After removing duplicates, a preliminary screening based on the article title and abstract was conducted and then 79 studies remained for assessment. According to the PRISMA 2020 (12), articles were screened by reading the full text, and excluding those without full-text availability, inconsistent trial designs, and conference papers. Finally, a total of 19 articles were included, involving 1,488 cases. For details, please refer to the Supplementary Figure 1.

### 3.2 Participant and intervention characteristics

Among the 19 studies that were finally included, 9 trials employed yoga intervention (17–25). Among the traditional Chinese exercise, 6 involved Qigong (26–31), 3 involved Baduanjin (32–34), and one involved Tai Chi (35). There were 11 studies (17, 19–22, 24–27, 32, 35) conducted on breast cancer patients, 2 on lymphoma (18, 31), 1 on lung cancer (28), 1 on nasopharyngeal carcinoma (33), 1 on colon cancer, and 3 involving patients with various other types of cancers (23, 29, 30). The included studies are summarized in Table 2.

**Table 2:** Characteristics of participants and interventions.

Source	Country (language)	Cancer type	Treatment phase	Intervention dose			Age	Intervening measure	Simple size	Assessment scale
				Session length	Frequency	Total weeks				
Chandwani et al. (17)	US (English)	Breast	During radiotherapy	60 min/session	up to 3 sessions/week	6	T: 52.38 ± 1.35 C: 52.11 ± 1.34	T: Yoga C: Wait-list	T: 53 C: 54	F: BFI
Chang et al. (26)	China (English)	Breast	During chemotherapy	35 min/day	5 days/week	15	T: 64.29 ± 6.71 C: 52.77 ± 8.53	T: Qigong C: No Qigong practice	T: 30 C: 30	F: EORTC QLQ-C30 Fatigue H: EORTC QLQ-C30 Global health
Chen et al. (27)	China (English)	Breast	During radiotherapy	ND	1 session/week	5	T: 45.3 ± 6.30 C: 44.7 ± 9.70	T: Qigong C: Wait-list	T:49 C:47	D: CES-D; F: BFI S: PSQI H: FACT-G
Cohen et al. (18)	US (English)	Lymphoma	During treatment or within 12 months after completion of treatment	ND	1 session/week	7	ND	T: Yoga C: Wait-list	T: 19 C: 19	D: CES-D F: BFI
Cramer et al. (19)	Germany (English)	Breast	Survivorship	90 min/session	1 session/week	12	T: 48.3 ± 4.80 C: 50.0 ± 6.70	T: Yoga C: Usual care	T: 19 C: 21	A: HADS-A D: HADS-D
Eyigör et al. (20)	Turkey (English)	Breast	Survivorship	ND	2 sessions/week	10	T: 51.40 ± 10.60 C: 50.7 ± 7.60	T: Yoga C: No yoga intervention reported	T: 15 C: 16	H: EORTC QLQ-C30 Global health
Kiecolt-Glaser et al. (21)	US (English)	Breast	Survivorship	90 min/session	2 sessions/week	12	ND	T: Yoga C: Wait-list	T: 100 C: 100	D: CES-D
Liu et al. (22)	China (English)	Breast	During adjuvant chemotherapy	90 min/session	1 session/week	8	T: 51.30 ± 12.80 C: 52.10 ± 11.60	T: Yoga C: Usual care	T: 68 C: 68	A: HADS-A; D: HADS-D; H: FACT-B
Molassiotis et al. (28)	Vietnam (English)	Lung	Survivorship/post-treatment	90 min/session, 2 sessions/week for 2 weeks, then home practice ≥30 min/day, 5 days/week for 4 weeks		6	T: 57.62 ± 9.63 C: 56.06 ± 9.25	T: Qigong C: Usual care	T: 30 C: 30	H: EORTC QLQ-C30 Global health
Namazinia et al. (23)	Iran (English)	Various	During chemotherapy	20–30 min/session	1 session/week	4	T: 49.00 ± 9.60 C: 45.20 ± 12.60	T: Yoga C: Usual care	T: 34 C: 35	F: EORTC QLQ-C30 Fatigue H: EORTC QLQ-C30 Global health
Oh et al. (29)	Australia (English)	Various	During treatment/early survivorship	90 min/session	2 sessions/week	10	T: 64.60 ± 12.30 C: 61.10 ± 11.00	T: Qigong C: Usual care	T: 23 C: 31	H: FACT-G
Oh et al. (30)	Australia (English)	Various	During chemotherapy/post-chemotherapy	90 min/session	2 sessions/week	10	T: 60.10 ± 11.70 C: 59.90 ± 11.30	T: Qigong C: Usual care	T: 54 C: 54	H: FACT-G
Ratcliff et al. (24)	US (English)	Breast	During radiotherapy	60 min/session	up to 3 sessions/week	6	T: 52.38 ± 8.85 C: 52.11 ± 9.09	T: Yoga C: Usual care	T: 43 C: 46	D: CES-D; S: PSQI
Vadiraja et al. (25)	India (English)	Breast	During radiotherapy	60 min/session	Daily	6	ND	T: Yoga C: Brief supportive therapy	T: 42 C: 33	F: EORTC QLQ-C30 Fatigue
Vargas-Román et al. (31)	Spain (English)	Lymphoma	Survivorship	60 min/session	2 sessions/week	8	T: 45.20 ± 10.88 C: 43.74 ± 10.53	T: Qigong C: Health habit suggestions	T: 20 C: 19	A: HADS-A; D: HADS-D
Wei et al. (32)	China (English)	Breast	During chemotherapy	30 min/session	5 sessions/week	12	ND	T: Baduanjin C: Usual care	T: 32 C: 35	A: HADS-A; D: HADS-D; H: FACT-B
Wen et al. (33)	China (English)	Nasopharynx carcinoma	Survivorship/after chemoradiotherapy	40 min/day	5 days/week	12	T: 45.55 ± 8.99 C: 47.07 ± 9.43	T: Baduanjin C: Usual care	T: 36 C: 39	H: FACT-G; S: PSQI
Wu et al. (34)	China (Chinese)	Colon Cancer	During chemotherapy	30 min/session	1 session/day	8	ND	T: Baduanjin C: Usual care	T: 15 C: 15	S: PSQI
Yao et al. (35)	China (English)	Breast	During adjuvant chemotherapy	60 min/session	2 sessions/week	8	T: 45.3 ± 8.50 C: 48.6 ± 7.80	T: TaiChi C: Usual care	T: 36 C: 33	D: HADS-D; S: PSQI; H: FACT-B

T, Experimental group; C, Control group; A, Anxiety; D, Depression; F, Fatigue; S, Sleep quality; H, HRQOL; HADS-A, Hospital Anxiety and Depression Scale—Anxiety subscale; HADS-D, Hospital Anxiety and Depression Scale—Depression subscale; CES-D, Center for Epidemiologic Studies Depression Scale; BFI, Big Five Inventory; EORTC, QLQ-C30 European Organization for Research and Treatment of Cancer Quality of Life Questionnaire—Core 30; PSQI, Pittsburgh Sleep Quality Index; HRQOL, Health-Related Quality of Life; FACT-B, Functional Assessment of Cancer Therapy—Breast cancer; FACT-G, Functional Assessment of Cancer Therapy - General; ND, Not-described.

### 3.3 Risk of bias assessment

The ROB 2.0 was used for quality assessment of the 19 included RCTs, and evaluated from five aspects of bias, including arising from the randomization process, deviations from intended intervention, missing outcome data, measurement of the outcome, and bias in selection of the reported result (Domain 1–5). Overall risk-of-bias judgments were derived according to the ROB 2.0 algorithm: studies were rated as “low risk” only if all domains were low risk; “high risk” if at least one domain was rated high risk; otherwise, they were rated as “some concerns.” The assessment results show that in Domain 1 (D1), 12 studies were rated as low-risk, and 7 studies as some concerns. For D2, 7 with high, 10 studies had some concerns, and 2 studies had low-risk. In D3, 5 were in high-risk and the other studies were shown low. In D4, 14 studies indicated some concerns and 5 studies were rated as low risk. Finally, in D5, 1 study indicated high-risk, 15 studies were some concerns, and 3 studies had low. In general, through methodological quality assessment, 15 studies were judged as having some concerns, 4 as high risk, and no study rated as low risk overall. This is due to the trial design. All participants and intervention subjects in the included studies were aware of their assigned groups. And the measurement method of the research results was through scales, so the outcome indicators were greatly influenced by the subjective factors of the patients. Although the blind design in behavioral intervention trials is challenging, these limitations may still affect the reliability of the results. Supplementary Figures 2, 3 depict quality evaluation charts.

### 3.4 Results of syntheses

#### 3.4.1 Significant improvement in average anxiety levels

A total of four researches involving 281 participants were included for the anxiety outcome. The outcome measure was measured using the HADS-A scale, and the scores were positively correlated with the anxiety levels of the patient. The results suggested that mind–body exercise compared with the control group significantly demonstrated reduction in the anxiety levels [SMD = −0.39, 95% CI (−0.65, −0.13), *p* = 0.004]. The heterogeneity of the studies was low (*I*^2^ = 14%), suggesting the results was relatively robust.

Subgroup analyses investigated whether the effects of mind–body exercise on anxiety varied depending on the intervention type and duration. By intervention type, yoga did not show a statistically significant effect on anxiety [SMD = −0.23, 95% CI (−0.72, 0.27), *p* = 0.37], whereas traditional Chinese exercise showed a significant reduction [SMD = −0.55, 95% CI (−0.94, −0.17), *p* = 0.005]. However, the subgroup difference test was not significant (*x*^2^ = 1.05, *p* = 0.31). Duration-based subgroup analyses for anxiety did not show significant effects in either subgroup, and no subgroup differences were detected (Supplementary Figure 4).

#### 3.4.2 Significant improvement in average depression levels

A total of nine studies involving 765 patients were included. The study used the HADS-D and CES-D scales to assess the outcomes, higher scores indicated worse depression level/symptom. The results of meta-analyses showed that after the intervention, the mind–body exercise group significantly reduced depression compared with the control [SMD = −0.34, 95%CI (−0.59, −0.10), and *p* = 0.007].

The research was grouped based on the intervention methods. This subgroup analysis indicates that traditional Chinese exercise reduced the depression levels of cancer patients, and this difference was statistically significant [SMD = −0.24, 95%CI (−0.42, −0.07), *p* = 0.007]. In contrast, the improvement trends observed in the yoga group did not achieve statistically significant [SMD = −0.52, 95%CI (−1.09, 0.05), *p* = 0.08]. The test for subgroup difference was not significant (*x*^2^ = 0.81, *p* = 0.37).

The intervention duration was divided into two groups: <8 weeks and ≥8 weeks. The reduction effect of ≥8 weeks was significantly significant [<8 weeks: SMD = 0.01, 95% CI (−0.26, 0.27), *p* = 0.96; and the ≥8 weeks: SMD = −0.50, 95% CI (−0.80, −0.20), *p* = 0.0009]. The test of the subgroup difference was significant (*x*^2^ = 6.19, *p* = 0.01) (Supplementary Figure 5).

#### 3.4.3 Significant improvement in average fatigue levels

For fatigue, 437 patients (from six studies) were conducted. Fatigue was measured using the BFI and the EORTC QLQ-C30 Fatigue scales. The results showed that compared with usual care, the mind–body exercise group significantly reduced the fatigue scores [SMD = −0.40, 95% CI (−0.78, −0.02), *p* = 0.04].

Subgroup analysis was conducted based on different intervention methods. The fatigue score in the yoga group showed a significant decrease [SMD = −0.63, 95% CI (−0.97, −0.30), *p* = 0.0002, *I*^2^ = 45%], while the traditional Chinese exercise group was not statistically significant [SMD = −0.01, 95% CI (−0.46, 0.44), *p* = 0.97, *I*^2^ = 49%]. The test for subgroup differences was statistically significant (*x*^2^ = 4.75, *p* = 0.03), indicating that intervention type may contribute to between-study differences; however, this finding should be interpreted cautiously given the smaller number of studies in some subgroups. The duration-based subgroup analyses showed no statistically significant effect in either subgroups and no subgroup differences were detected (Supplementary Figure 6).

#### 3.4.4 Significant improvement in average sleep quality levels

Sleep quality was assessed using the PSQI scale, with lower scores indicating better sleep. A meta-analysis of five studies (*n* = 359) showed that mind–body exercise significantly improved sleep quality compared with usual care [SMD = −0.60, 95% CI (−1.15, −0.05), *p* = 0.03]. However, heterogeneity was substantial (*I*^2^ = 84%), and the pooled estimate should therefore be interpreted cautiously.

To explore potential sources of heterogeneity, subgroup analyses were performed using intervention duration. The <8 weeks subgroup showed no significant effect [SMD = 0.01, 95% CI (−0.34, 0.35), *I*^2^ = 30%, *p* = 0.97], whereas the ≥8 weeks subgroup demonstrated a significant improvement in sleep quality [SMD = −1.02, 95% CI (−1.56, −0.48), *I*^2^ = 61%, *p* = 0.0002]. The test for subgroup differences was statistically significant (*x*^2^ = 9.97, *p* = 0.002), suggesting that intervention duration may be associated with different effect estimates. Nevertheless, because subgroup analyses were exploratory and heterogeneity remained moderate within the ≥8 weeks subgroup, these findings should be interpreted cautiously.

When stratified by intervention type, yoga (single study) did not show a significant effect [SMD = −0.17, 95% CI (−0.59, 0.24), *p* = 0.41], whereas traditional Chinese exercise showed a significant improvement [SMD = −0.74, 95% CI (−1.46, −0.02), *I*^2^ = 87%, *p* = 0.04]. The test for subgroup differences was not significant (*x*^2^ = 1.77, *p* = 0.18), indicating insufficient evidence that effects differ by intervention type (Supplementary Figure 7).

#### 3.4.5 Significant improvement in average health-related quality of life levels

This outcome included 11 studies with 879 patients. These studies reported the HRQOL—related results of the patients, which were evaluated using the FACT-B, FACT-G, and EORTC QLQ-C30 Global Health scales, higher total scores indicate better HRQOL. In the results, compared with the usual care, mind–body exercise significantly increased the HRQOL levels [SMD = 0.38, 95% CI (0.17, 0.60), *p* = 0.0005].

Subgroup analyses were conducted according to intervention type. The yoga subgroup showed a borderline improvement in HRQOL [SMD = 0.26, 95% CI (0.00, 0.51), *p* = 0.05, *I*^2^ = 0%], whereas the traditional Chinese exercise subgroup demonstrated a significant improvement [SMD = 0.45, 95% CI (0.17, 0.73), *p* = 0.002, *I*^2^ = 67%]. However, the test for subgroup differences was not statistically significant (*x*^2^ = 1.01, *p* = 0.32).

When stratified by intervention duration, the ≥8 weeks subgroup showed a significant improvement in HRQOL [SMD = 0.50, 95% CI (0.29, 0.72), *p* < 0.00001, *I*^2^ = 39%], whereas the <8 weeks subgroup did not reach statistical significance [SMD = 0.10, 95% CI (−0.31, 0.52), *p* = 0.62, *I*^2^ = 67%]. The test for subgroup differences was not statistically significant (*x*^2^ = 2.75, *p* = 0.10) (Supplementary Figure 8).

### 3.5 Sensitivity analysis and publication bias

This study conducted sensitivity analyses to assess the robustness of the comprehensive research results. Among these five outcomes, the robustness of anxiety was low: when individual trials were excluded and removal of high-risk studies changed the statistical significance. Depression showed the highest robustness. In the fatigue outcome, the robustness was also at a medium level: the direction of the effect was maintained, but the statistical significance did not remain completely stable in the leave-one-out approach. The robustness of sleep quality was at a medium level, and its statistical significance was sensitive to the exclusion of specific studies, and the heterogeneity remained high is all analyses, so results should be interpreted with caution. Finally, HRQOL indicators showed moderate robustness. When high-risk trials were excluded, the summary estimates weakened and affected the statistical significance (Supplementary Table 2).

The publication-bias of the five outcome indicators was mainly evaluated through visual inspection of the funnel plot. Mild asymmetry was observed in some results with less than 10 studies (anxiety, depression, fatigue, and sleep quality); however, due to the limited number of trials, apart from qualitative evaluation, publication-bias could not be reliably assessed. For HRQOL involving 11 studies, this study conducted the Egger’s regression test, and found no evidence of a small-study effect (*p* = 0.870), indicating no obvious evidence of publication bias for the result (Supplementary Figure 9).

### 3.6 Certainty of evidence

In the GRADE evaluation results, anxiety and HRQOL were rated as low; depression and fatigue were rated as moderate; and the certainty for sleep quality was rated as very low. For detailed information, please refer to Supplementary Table 3 in the attachment.

## 4 Discussion

A systematic review and meta-analysis of RCTs demonstrated that, compared with the controls, mind–body exercise effectively reduced anxiety, depression, and fatigue levels, and also improved sleep quality and HRQOL. Mind–body exercise may be a feasible adjunctive approach for symptom management in cancer patients.

Prior reviews indicate that mind–body exercise effects may vary by dose/duration, intervention type, comparator choice, and outcome measurement. To distinguish statistical from clinical significance, this study compared pooled effect sizes with published minimal clinically important differences (MCIDs) where available. Benefits for anxiety/depression appear modest relative to commonly cited HADS thresholds, whereas effects for fatigue and HRQOL are more often within ranges considered clinically meaningful on common cancer instruments (36–39). For sleep quality, potential clinical relevance should be interpreted cautiously given variable PSQI MCID estimates and substantial heterogeneity (38).

### 4.1 Impact on mental health

Cancer patients often have psychological problems such as anxiety and depression. The prevalence of anxiety disorders is approximately 18–20%, while that of depression is about 12–21% (1). The meta-analysis showed significant improvements with mind–body exercise; however, their clinical relevance should be interpreted in light of effect magnitude, outcome scales, and the certainty of evidence (40). Subgroup analyses suggested potentially different effect estimates by intervention type; however, subgroup findings were exploratory and tests for subgroup differences were not consistently significant, and should be interpreted cautiously. The study by Sun et al. (41) is consistent with the findings of this study. Modern research has shown that traditional Chinese exercise can regulate heart rate variability (HRV) by improving the balance of the autonomic nervous system, that is, by enhancing the activity of the parasympathetic/vagal nerve and reducing sympathetic nerve tension, thereby achieving the effects of emotional regulation and stress reduction (42, 43). The results regarding yoga are different from previous studies (44). This may be due to the lack of research in yoga subgroup, resulting in limited statistical capacity. Moreover, differences in intervention modality, intervention duration, and cancer type across the included yoga trials may have reduced statistical power and precision, thereby lowering the certainty and generalizability of the pooled estimate; therefore, the yoga-related findings should be interpreted cautiously. The study quality also related to the quality of inclusion in the literature. Meanwhile, long-term interventions (lasting for ≥8 weeks) were associated with reductions in depression levels, consistent with existing research (40).

### 4.2 Impact on physical symptoms and quality of life

Cancer patients are often troubled by sleep disorders and fatigue (45). The results of this study found that mind–body exercise can significantly alleviate the fatigue and improve the HRQOL. The improvements in sleep quality were also suggested to be effective, but the heterogeneity was relatively high. These results were consistent with the existing studies (46, 47). These benefits can be partly explained by biological mechanisms: mind–body exercise reduced pro-inflammatory cytokines, restored the hypothalamic–pituitary–adrenal (HPA) axis homeostasis, enhanced the parasympathetic system (increase HRV), and regulated sleep-related pathways such as melatonin, thereby simultaneously improved sleep and fatigue and thereby influenced HRQOL (48, 49).

In subgroup analyses, when grouped by intervention time, an intervention lasting for ≥8 weeks was effective in improving sleep quality, and HRQOL. Exploratory subgroup analyses suggested that yoga showed clearer signals for fatigue, whereas traditional Chinese exercise showed more consistent signals for psychological and sleep-related outcomes; these observations are hypothesis-generating rather than definitive comparative conclusions.

### 4.3 Limitations of research and trends in future

The limitations of this study are listed below. First, among the 19 studies, no study was rated as low risk overall, 15 indicated with some concerns, and 4 as high risk of bias. This may affect the strength of clinical evidence. Second, none of the studies used blinding method, which have affected the reliability of the results. Third, all the outcome indicators were evaluated using scales, although the tools were reliable, their self-reporting nature was subjective. Fourth, publication bias could not be reliably ruled out. Visual inspection of funnel plots suggested possible small-study effects, and the Egger’s test was only feasible for HRQOL due to the limited number of studies for other outcomes; therefore, any assessment of publication bias should be interpreted cautiously. Fifth, due to the different intervention methods, cancer types and measurement scales included in the trial, complicated the interpreted results. Finally, the GRADE assessment indicated that the certainty of evidence ranged from very low to moderate across outcomes, mainly downgraded for study limitations, imprecision, and inconsistency, which should be considered when interpreting these findings. The subgroup analyses of the study were exploratory and were not registered in advance, these results should be interpreted with caution and regarded as research findings that help to generate hypotheses.

Mind–body exercise should be considered as a feasible non-pharmacological treatment option for managing cancer symptoms in clinical practice. By combining body relaxing, controlling breathing, and performing gentle movements, may help in maintaining emotional stability, alleviate fatigue and insomnia, and improve HRQOL. Future research and implementation work should examine strategies to improve accessibility and adherence to mind–body exercise programs (e.g., standardized instructor training, supervised delivery models, and integration into routine supportive care), while considering feasibility and potential reimbursement pathways. Clinical trials should recruit more diverse samples and include objective physiological outcomes (e.g., cortisol and inflammatory biomarkers) to strengthen the evidence base and support standardized mind–body exercise protocols.

## 5 Conclusion

In this article, mind–body exercise, as a non-drug supplementary intervention for managing symptoms in patients with cancer, depicts great potential to alleviate mental health, physical symptoms, and HRQOL. The analysis shows that this exercise has statistically significant advantages in relieving anxiety, depression, fatigue, and HRQOL. Although sleep problems also improved, this improvement was accompanied by high heterogeneity. The subgroup analysis showed that long-term interventions (≥ 8 weeks) significantly improved symptoms except for anxiety and fatigue. Traditional Chinese exercise may be more effective in improving anxiety, depression, and sleep quality. Patients with severe fatigue could choose yoga to help relieve their symptoms. Both yoga and traditional Chinese exercise can improve patients’ quality of life. The research emphasized the important, comprehensive role of mind–body exercise in cancer management, revealing its benefits in regulating emotions, reducing physical discomfort, and improving HRQOL. It provided guidance for non-pharmacological complementary therapies and recommends including mind–body exercise in cancer treatment plans, offering new ideas for the long-term symptom management of patients with cancer.

## Data Availability

The original contributions presented in the study are included in the article/Supplementary material, further inquiries can be directed to the corresponding author.
