# Supplementary material for: Mind–body exercise for symptom management in cancer: a systematic review and meta-analysis
Source: Front Public Health. 2026 Mar 20;14:1762140. doi: 10.3389/fpubh.2026.1762140 (PMC13046479; doi:10.3389/fpubh.2026.1762140)
Supplement: Supplementary file 1 [file data_sheet_1.pdf]

Supplementary Figures

1 Supplementary Figure 1

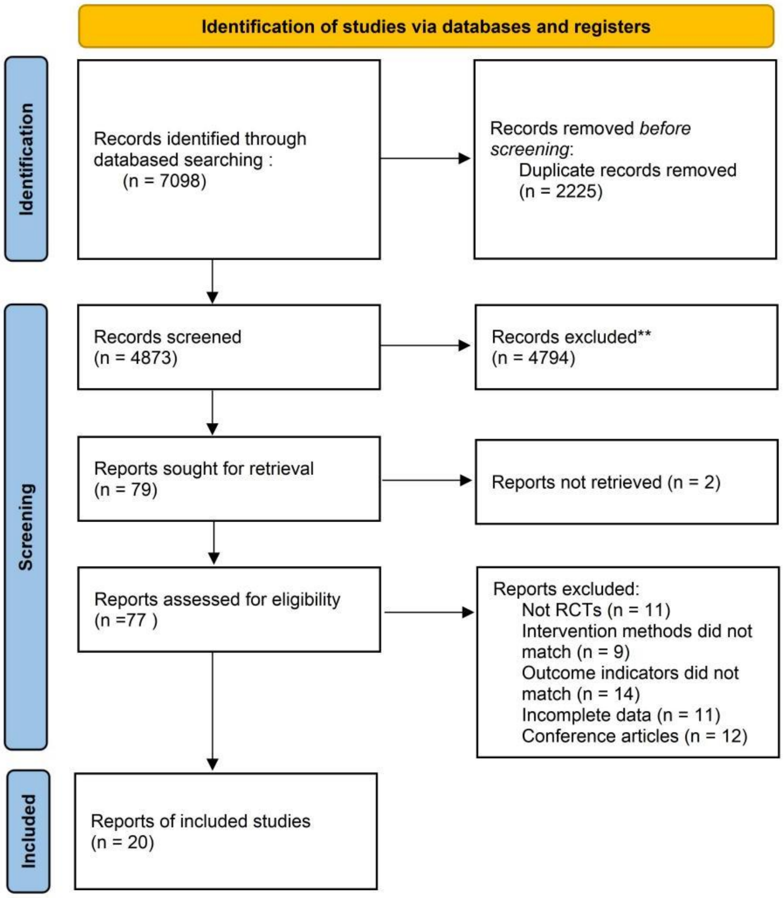

Supplementary Figure 1. Flow diagram of systematic literature search.

2 Supplementary Figure 2

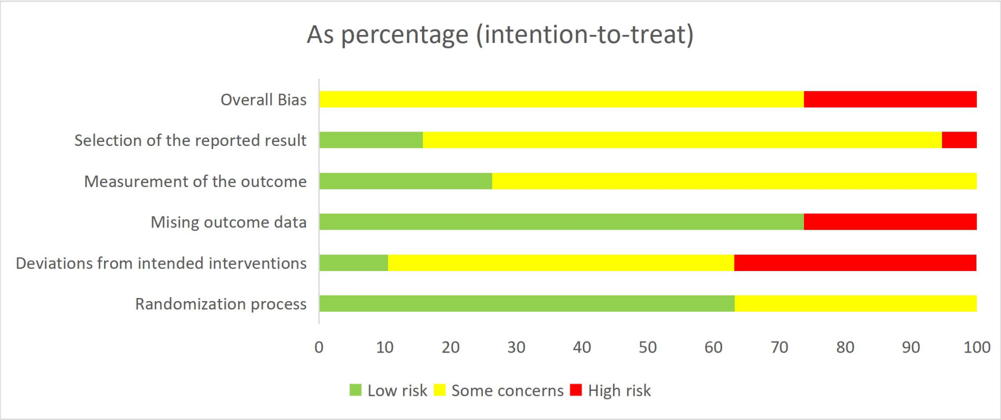

Supplementary Figure 2. Quality evaluation distribution chart.

3 Supplementary Figure 3

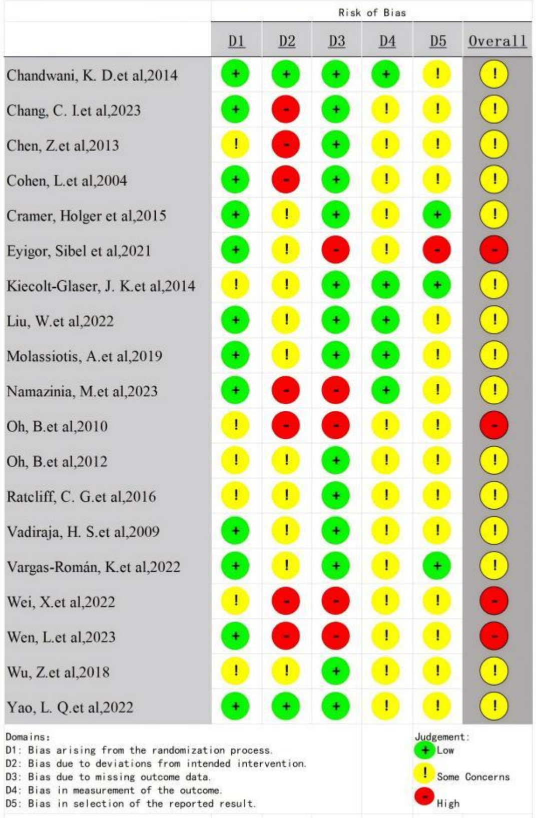

Supplementary Figure 3. Quality evaluation summary chart.

4 Supplementary Figure 4

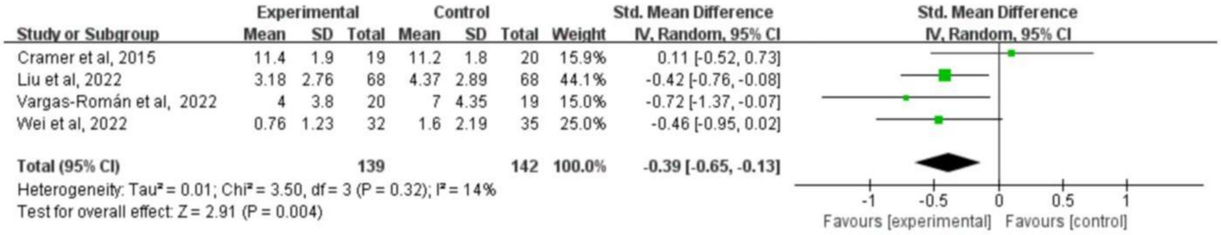

A

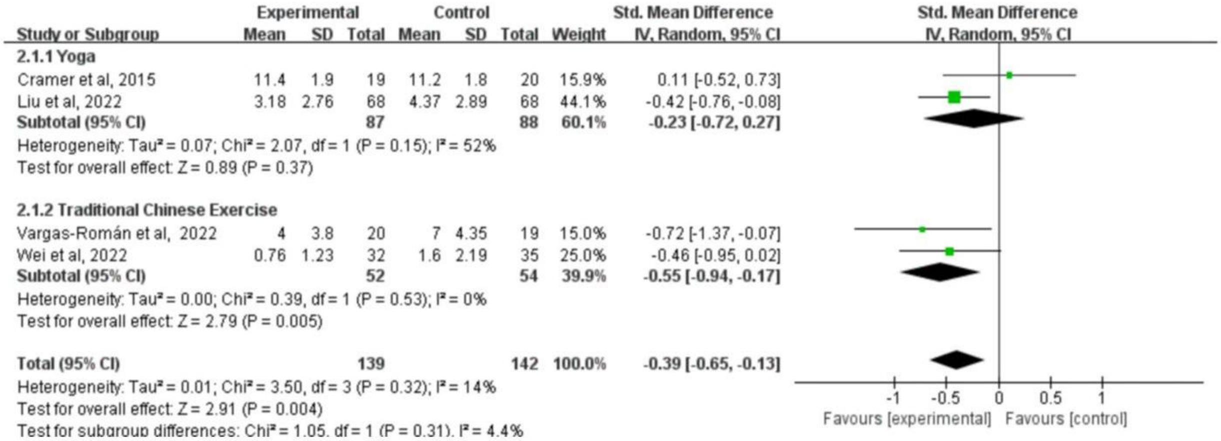

B

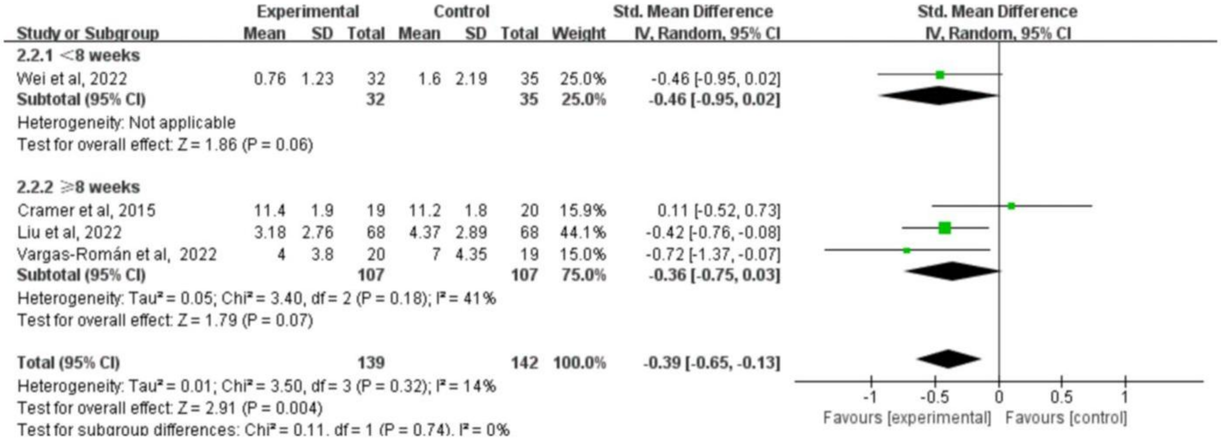

C

Supplementary Figure 4. (A) Forest plot forest plot of the effects of mind-body exercise on anxiety. (B) Subgroup analysis based on intervention type. (C) Subgroup analysis based on intervention time.

5 Supplementary Figure 5

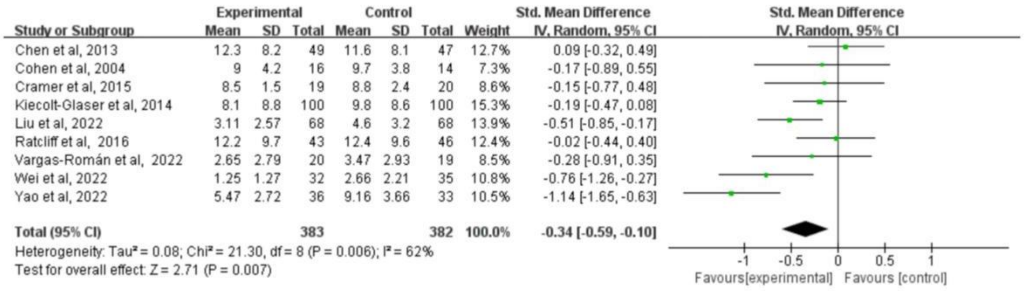

A

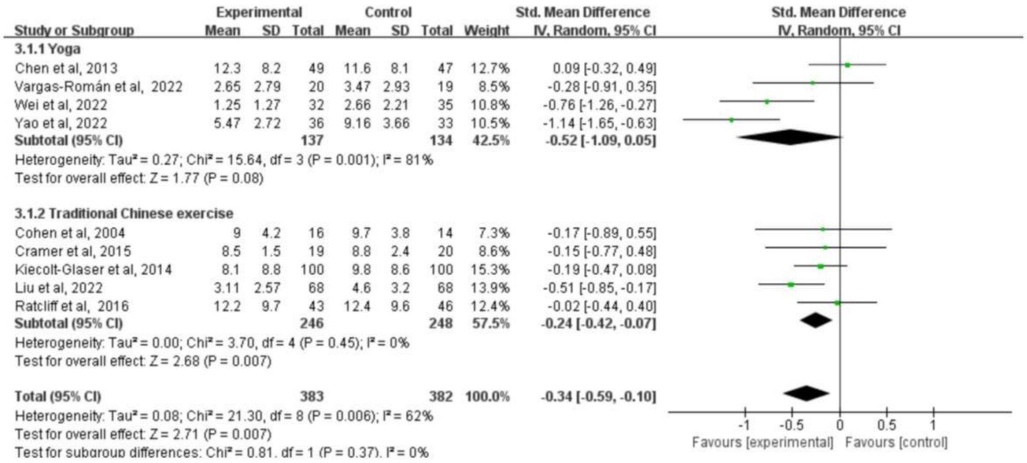

B

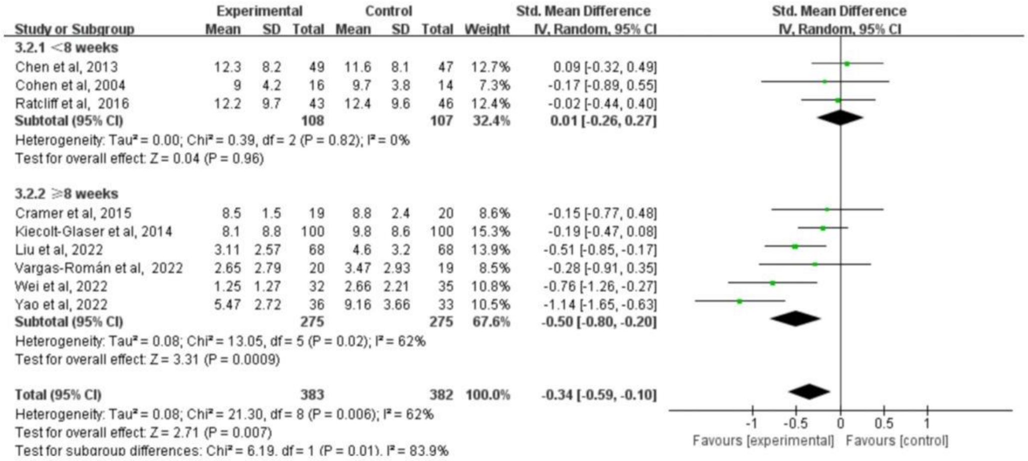

C

Supplementary Figure 5. (A) Forest plot forest plot of the effects of mind-body exercise on depression. (B) Subgroup analysis based on intervention type. (C) Subgroup analysis based on intervention time.

6 **Supplementary Figure 6**

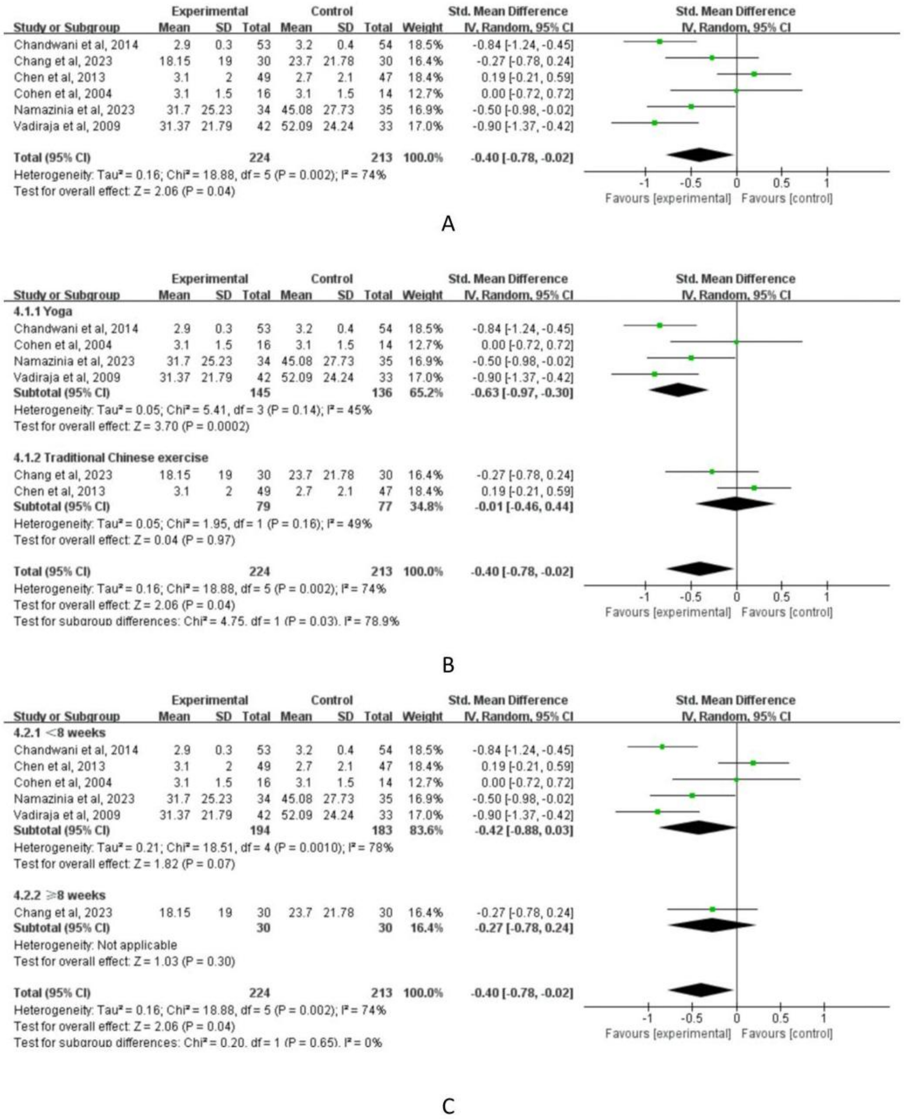

Supplementary Figure 6. (A) Forest plot forest plot of the effects of mind-body exercise on fatigue. (B) Subgroup analysis based on intervention type. (C) Subgroup analysis based on intervention time.

7 Supplementary Figure 7

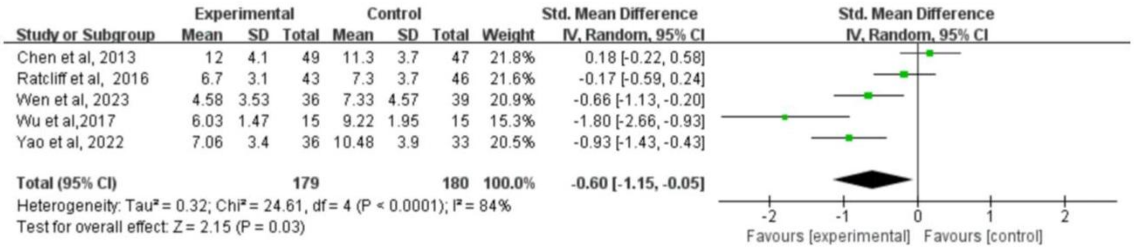

A

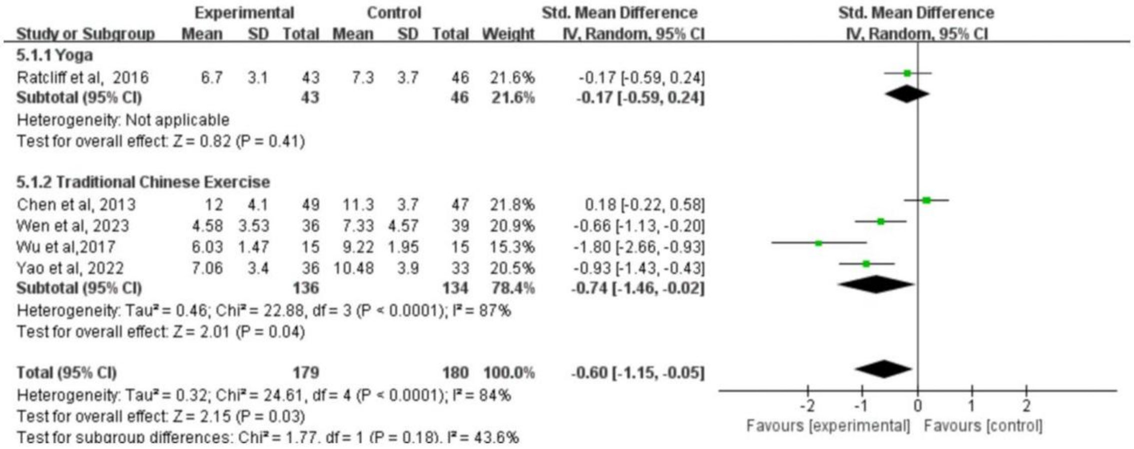

B

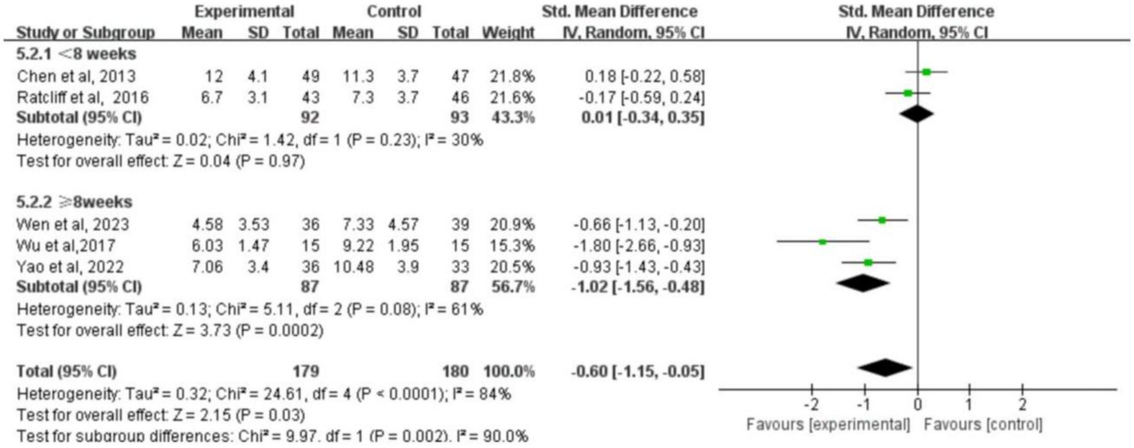

C

Supplementary Figure 7. (A) Forest plot forest plot of the effects of mind-body exercise on sleep quality. (B) Subgroup analysis based on intervention time.

8 Supplementary Figure 8

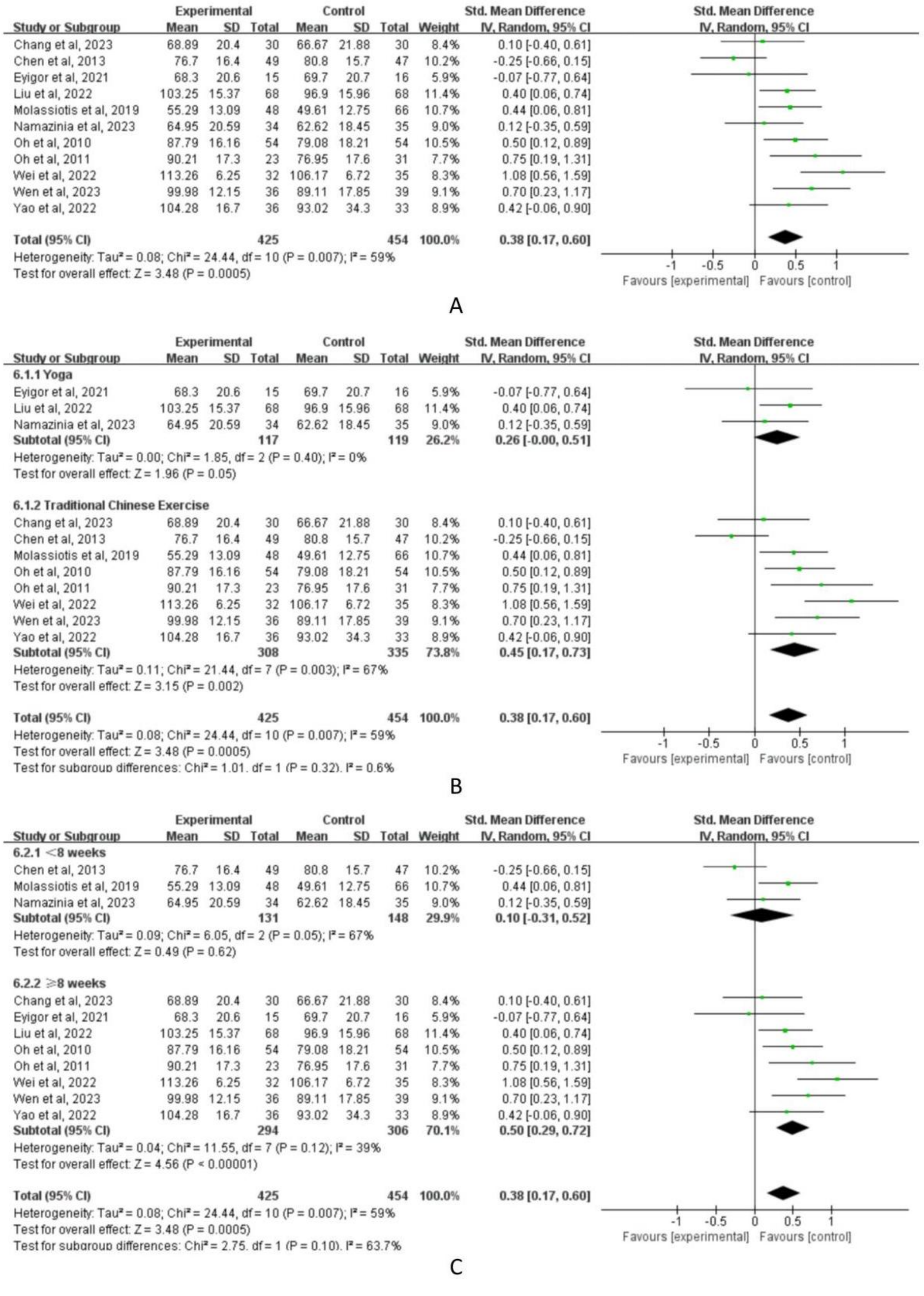

Supplementary Figure 8. (A) Forest plot forest plot of the effects of mind-body exercise on HRQOL. (B) Subgroup analysis based on intervention type. (C) Subgroup analysis based on intervention time.

9 Supplementary Figure 9

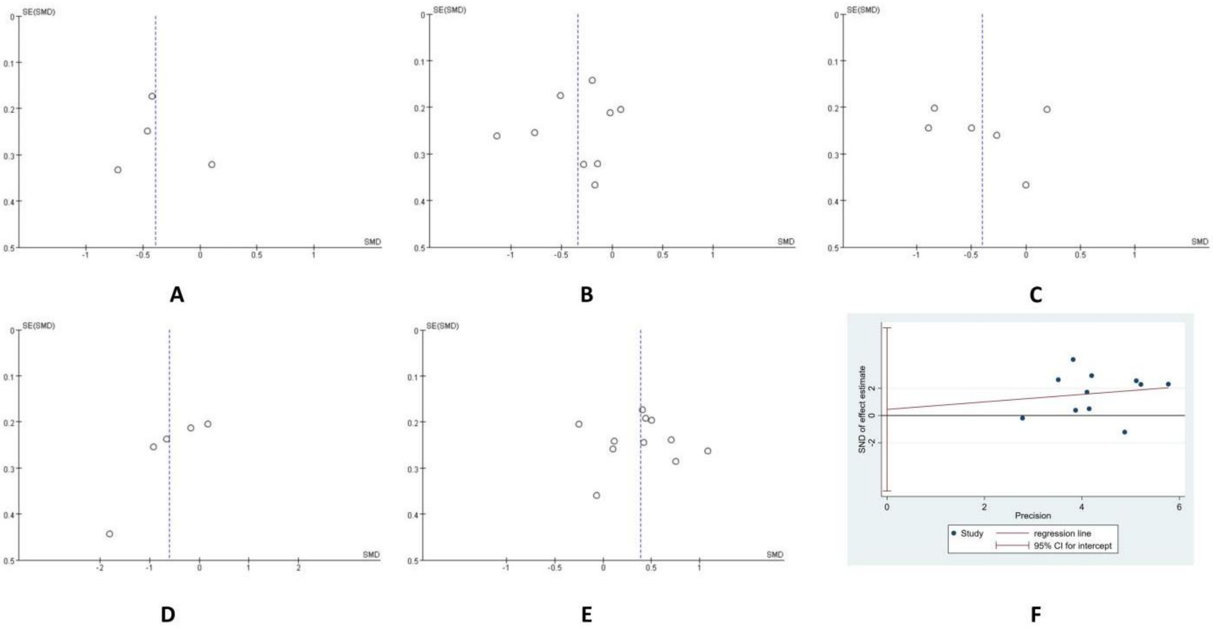

Supplementary Figure 9. Funnel plots: (A) Anxiety; (B) Depression; (C) Fatigue; (D) Sleep Quality; (E) HRQOL; (F) Egger's test plot for HRQOL.
