# Supplementary material for: Mind–body exercise for symptom management in cancer: a systematic review and meta-analysis
Source: Front Public Health. 2026 Mar 20;14:1762140. doi: 10.3389/fpubh.2026.1762140 (PMC13046479; doi:10.3389/fpubh.2026.1762140)
Supplement: Supplementary file 2 [file data_sheet_2.pdf]

Supplementary Tables

1. Supplementary Table 1

Databases	Search strategy
PubMed	#1 ((((((((((traditional Chinese exercise[Title/Abstract]) OR (Chinese traditional exercise[Title/Abstract])) OR (mind body[Title/Abstract])) OR (mind exercise[Title/Abstract])) OR ((((((((((((\"Tai Ji\"[Mesh]) OR (Tai Ji[Title/Abstract])) OR (Tai-ji[Title/Abstract])) OR (Tai Chi[Title/Abstract])) OR (Chi, Tai[Title/Abstract])) OR (Tai Chi Chuan[Title/Abstract])) OR (Taiji[Title/Abstract])) OR (Taijiquan[Title/Abstract])) OR (T'ai Chi[Title/Abstract])) OR (Tai Ji Quan[Title/Abstract])) OR (Ji Quan, Tai[Title/Abstract])) OR (Quan, Tai Ji[Title/Abstract]))) OR ((((\"Qigong\"[Mesh]) OR (Qigong[Title/Abstract])) OR (Ch'i Kung[Title/Abstract])) OR (Qi Gong[Title/Abstract]))) OR (baduanjin[Title/Abstract])) OR (yijinjing[Title/Abstract])) OR (liuzijue[Title/Abstract])) OR (wuqinxi[Title/Abstract])) OR ((((((\"Yoga\"[Mesh]) OR (Yoga[Title/Abstract])) OR (pranayama[Title/Abstract])) OR (asana*[Title/Abstract])) OR (dhyana[Title/Abstract])) OR (pilates[Title/Abstract]))<br>#2 (((((((((((((((((\"Neoplasms\"[Mesh]) OR (Neoplasms[Title/Abstract])) OR (Tumors[Title/Abstract])) OR (Neoplasia[Title/Abstract])) OR (Neoplasias[Title/Abstract])) OR (Neoplasm[Title/Abstract])) OR (Tumor[Title/Abstract])) OR (Cancer[Title/Abstract])) OR (Cancers[Title/Abstract])) OR (Malignant Neoplasm[Title/Abstract])) OR (Malignancy[Title/Abstract])) OR (Malignancies[Title/Abstract])) OR (Malignant Neoplasms[Title/Abstract])) OR (Neoplasm, Malignant[Title/Abstract])) OR (Neoplasms, Malignant[Title/Abstract])) OR (Benign Neoplasms[Title/Abstract])) OR (Neoplasms, Benign[Title/Abstract])) OR (Neoplasm, Benign[Title/Abstract])) OR (Benign Neoplasm[Title/Abstract])<br>#3 (((((((((((\"Anxiety\"[Mesh]) OR (Anxiety[Title/Abstract])) OR (Angst[Title/Abstract])) OR (Nervousness[Title/Abstract])) OR (Hypervigilance[Title/Abstract])) OR (Social Anxiety[Title/Abstract])) OR (Anxieties, Social[Title/Abstract])) OR (Anxiety, Social[Title/Abstract])) OR (Social Anxieties[Title/Abstract])) OR (Anxiousness[Title/Abstract])) OR (((((((\"Depression\"[Mesh]) OR (Depression[Title/Abstract])) OR (Depressive Symptoms[Title/Abstract])) OR (Depressive Symptom[Title/Abstract])) OR (Symptom, Depressive[Title/Abstract])) OR (Emotional Depression[Title/Abstract])) OR (Depression, Emotional[Title/Abstract]))) OR ((((((\"Quality of Life\"[Mesh]) OR (Quality of Life)) OR (Life Quality)) OR (Health-Related Quality Of Life)) OR (Health Related Quality Of Life)) OR (HRQOL))<br>#4 #1 AND #2 AND #3
Embase	#1 'traditional chinese exercise'/exp OR 'mind body exercise'/exp OR 'tai chi'/exp OR 'qigong'/exp OR 'yoga'/exp OR 'pilates'/exp OR 'chinese traditional exercise':ti,ab,kw OR 'traditional chinese exercise':ti,ab,kw OR 'mind body exercise':ti,ab,kw OR 'mind body':ti,ab,kw OR 'mind exercise':ti,ab,kw OR 'tai chi chuan':ti,ab,kw OR 'tai ji':ti,ab,kw OR 'tai ji

	quan':ti,ab,kw OR 'taiji':ti,ab,kw OR 'taijiquan':ti,ab,kw OR 'tai chi':ti,ab,kw OR 'chi kung':ti,ab,kw OR 'chigung':ti,ab,kw OR 'qi gong':ti,ab,kw OR 'qigong':ti,ab,kw OR 'baduanjin':ti,ab,kw OR 'yijinjing':ti,ab,kw OR 'liuzijue':ti,ab,kw OR 'wuqinxi':ti,ab,kw OR 'yogic meditation':ti,ab,kw OR 'yoga':ti,ab,kw OR 'pranayama':ti,ab,kw OR 'asana*':ti,ab,kw OR 'dhyana':ti,ab,kw OR 'pilates':ti,ab,kw<br>#2 'neoplasm'/exp OR 'acral tumor':ti,ab,kw OR 'acral tumour':ti,ab,kw OR 'neoplasia':ti,ab,kw OR 'neoplasms':ti,ab,kw OR 'neoplastic disease':ti,ab,kw OR 'neoplastic entity':ti,ab,kw OR 'neoplastic mass':ti,ab,kw OR 'tumor':ti,ab,kw OR 'tumoral entity':ti,ab,kw OR 'tumoral mass':ti,ab,kw OR 'tumorous entity':ti,ab,kw OR 'tumorous mass':ti,ab,kw OR 'tumors':ti,ab,kw OR 'tumour':ti,ab,kw OR 'tumoural entity':ti,ab,kw OR 'tumoural mass':ti,ab,kw OR 'tumourous entity':ti,ab,kw OR 'tumourous mass':ti,ab,kw OR 'tumours':ti,ab,kw OR 'neoplasm':ti,ab,kw OR 'malignant neoplasm'/exp OR 'cancer':ti,ab,kw OR 'cancers':ti,ab,kw OR 'malignant neoplasia':ti,ab,kw OR 'malignant neoplastic disease':ti,ab,kw OR 'malignant tumor':ti,ab,kw OR 'malignant tumour':ti,ab,kw OR 'neoplasia, malignant':ti,ab,kw OR 'neoplasmic malignancy':ti,ab,kw OR 'neoplastic malignancy':ti,ab,kw OR 'oncologic malignancy':ti,ab,kw OR 'oncological malignancy':ti,ab,kw OR 'tumor, malignant':ti,ab,kw OR 'tumoral malignancy':ti,ab,kw OR 'tumorous malignancy':ti,ab,kw OR 'tumour, malignant':ti,ab,kw OR 'malignant neoplasm':ti,ab,kw<br>#3 'depression'/exp OR 'major depression'/exp OR 'depressive disorder'/exp OR 'depressive episode'/exp OR 'depressive symptom'/exp OR 'clinical depression':ti,ab,kw OR 'central depression':ti,ab,kw OR 'depressive disease':ti,ab,kw OR 'depressive disorder':ti,ab,kw OR 'depressive episode':ti,ab,kw OR 'depressive illness':ti,ab,kw OR 'depressive personality disorder':ti,ab,kw OR 'depressive state':ti,ab,kw OR 'depressive symptom':ti,ab,kw OR 'depressive syndrome':ti,ab,kw OR 'depressivity':ti,ab,kw OR 'mental depression':ti,ab,kw OR 'parental depression':ti,ab,kw OR 'major depression':ti,ab,kw OR 'major depressive disorder':ti,ab,kw OR 'minor depression':ti,ab,kw OR 'depression':ti,ab,kw OR 'anxiety'/exp OR 'anxiety disorder'/exp OR 'generalized anxiety disorder'/exp OR 'anxiety':ti,ab,kw OR 'anxiety disorder':ti,ab,kw OR 'generalized anxiety disorder':ti,ab,kw OR 'anxious':ti,ab,kw<br>#4 #1 AND #2 AND #3
Cochrane Library	#1 (traditional Chinese exercise):ti,ab,kw OR (Chinese traditional exercise):ti,ab,kw OR (mind body):ti,ab,kw OR (mind exercise):ti,ab,kw OR MeSH descriptor: [Tai Ji] explode all trees OR (Tai Ji):ti,ab,kw OR (Tai-ji):ti,ab,kw OR (Tai Chi):ti,ab,kw OR (Chi, Tai):ti,ab,kw OR (Tai Chi Chuan):ti,ab,kw OR (Taiji):ti,ab,kw OR (Taijiquan):ti,ab,kw OR (T'ai Chi):ti,ab,kw OR (Tai Ji Quan):ti,ab,kw OR (Ji Quan, Tai):ti,ab,kw OR (Quan, Tai Ji):ti,ab,kw OR MeSH descriptor: [Qigong] explode all trees OR (Qigong):ti,ab,kw OR (Ch'i Kung):ti,ab,kw OR (Qi Gong):ti,ab,kw OR (baduanjin):ti,ab,kw OR (yijinjing):ti,ab,kw OR (liuzijue):ti,ab,kw OR (wuqinxi):ti,ab,kw OR MeSH descriptor: [Yoga] explode all trees OR (Yoga):ti,ab,kw OR (pranayama):ti,ab,kw OR (asana*):ti,ab,kw OR

	(dhyana):ti,ab,kw OR (pilates):ti,ab,kw<br>#2 MeSH descriptor: [Neoplasms] explode all trees OR (Neoplasms):ti,ab,kw OR (Tumors):ti,ab,kw OR (Neoplasia):ti,ab,kw OR (Neoplasias):ti,ab,kw OR (Neoplasm):ti,ab,kw OR (Tumor):ti,ab,kw OR (Cancer):ti,ab,kw OR (Cancers):ti,ab,kw OR (Malignant Neoplasm):ti,ab,kw OR (Malignancy):ti,ab,kw OR (Malignancies):ti,ab,kw OR (Malignant Neoplasms):ti,ab,kw OR (Neoplasm, Malignant):ti,ab,kw OR (Neoplasms, Malignant):ti,ab,kw OR (Benign Neoplasms):ti,ab,kw OR (Neoplasms, Benign):ti,ab,kw OR (Neoplasm, Benign):ti,ab,kw OR (Benign Neoplasm):ti,ab,kw<br>#3 MeSH descriptor: [Anxiety] explode all trees OR (Anxiety):ti,ab,kw OR (Angst):ti,ab,kw OR (Nervousness):ti,ab,kw OR (Hypervigilance):ti,ab,kw OR (Social Anxiety):ti,ab,kw OR (Anxieties, Social):ti,ab,kw OR (Anxiety, Social):ti,ab,kw OR (Social Anxieties):ti,ab,kw OR (Anxiousness):ti,ab,kw OR MeSH descriptor: [Depression] explode all trees OR (Depression):ti,ab,kw OR (Depressive Symptoms):ti,ab,kw OR (Depressive Symptom):ti,ab,kw OR (Symptom, Depressive):ti,ab,kw OR (Emotional Depression):ti,ab,kw OR (Depression, Emotional):ti,ab,kw OR MeSH descriptor: [Quality of Life] explode all trees OR (Quality of Life) OR (Life Quality) OR (Health-Related Quality Of Life) OR (Health Related Quality Of Life) OR (HRQOL)<br>#4 #1 AND #2 AND #3
PsycINFO	#1 (traditional Chinese exercise) OR (Chinese traditional exercise) OR (mind body) OR (mind exercise) OR((Mesh=Tai Ji) OR (Tai Ji) OR (Tai-ji) OR (Tai Chi) OR (Chi, Tai) OR (Tai Chi Chuan) OR (Taiji) OR (Taijiquan) OR (T'ai Chi) OR (Tai Ji Quan) OR (Ji Quan, Tai) OR (Quan, Tai Ji)) OR ((Mesh=Qigong) OR (Qigong) OR (Ch'i Kung) OR (Qi Gong)) OR (baduanjin) OR (yijinjing) OR (liuzijue) OR (wuqinxi) OR ((Mesh=Yoga) OR (Yoga) OR (pranayama) OR (asana*) OR (dhyana) OR (pilates))<br>#2 ((Mesh=Neoplasms) OR (Neoplasms) OR (Tumors) OR (Neoplasia) OR (Neoplasias) OR (Neoplasm) OR (Tumor) OR (Cancer) OR (Cancers) OR (Malignant Neoplasm) OR (Malignancy) OR (Malignancies) OR (Malignant Neoplasms) OR (Neoplasm, Malignant) OR (Neoplasms, Malignant) OR (Benign Neoplasms) OR (Neoplasms, Benign) OR (Neoplasm, Benign) OR (Benign Neoplasm))<br>#3 ((Mesh=Anxiety) OR (Anxiety) OR (Angst) OR (Nervousness) OR (Hypervigilance) OR (Social Anxiety) OR (Anxieties, Social) OR (Anxiety, Social) OR (Social Anxieties) OR (Anxiousness)) OR ((Mesh=Depression) OR (Depression) OR (Depressive Symptoms) OR (Depressive Symptom) OR (Symptom, Depressive) OR (Emotional Depression) OR (Depression, Emotional)) OR ((Mesh=Quality of Life) OR (Quality of Life)) OR (Life Quality) OR (Health-Related Quality Of Life) OR (Health Related Quality Of Life) OR (HRQOL))<br>#4 #1 AND #2 AND #3
Web of Science	#1 (TI=((((((((((traditional Chinese exercise) OR (Chinese traditional exercise)) OR (mind body)) OR (mind exercise)) OR ((((((((((((Tai Ji) OR (Tai Ji)) OR (Tai-ji)) OR (Tai Chi)) OR (Chi, Tai)) OR (Tai Chi Chuan)) OR (Taiji)) OR (Taijiquan)) OR (T'ai Chi)) OR (Tai Ji Quan)) OR (Ji Quan,

Tai)) OR (Quan, Tai Ji))) OR ((((Qigong) OR (Qigong)) OR (Ch'i Kung)) OR (Qi Gong))) OR (baduanjin)) OR (yijinjing)) OR (liuzijue)) OR (wuqinxi)) OR ((((((Yoga) OR (Yoga)) OR (pranayama)) OR (asana*)) OR (dhyana)) OR (pilates)))) OR AB=(((((((((((traditional Chinese exercise) OR (Chinese traditional exercise)) OR (mind body)) OR (mind exercise)) OR (((((((((((Tai Ji) OR (Tai Ji)) OR (Tai-ji)) OR (Tai Chi)) OR (Chi, Tai)) OR (Tai Chi Chuan)) OR (Taiji)) OR (Taijiquan)) OR (T'ai Chi)) OR (Tai Ji Quan)) OR (Ji Quan, Tai)) OR (Quan, Tai Ji))) OR ((((Qigong) OR (Qigong)) OR (Ch'i Kung)) OR (Qi Gong))) OR (baduanjin)) OR (yijinjing)) OR (liuzijue)) OR (wuqinxi)) OR ((((((Yoga) OR (Yoga)) OR (pranayama)) OR (asana*)) OR (dhyana)) OR (pilates)))

#2 (TI=((((((((((((((((((Neoplasms) OR (Neoplasms)) OR (Tumors)) OR (Neoplasia)) OR (Neoplasias)) OR (Neoplasm)) OR (Tumor)) OR (Cancer)) OR (Cancers)) OR (Malignant Neoplasm)) OR (Malignancy)) OR (Malignancies)) OR (Malignant Neoplasms)) OR (Neoplasm, Malignant)) OR (Neoplasms, Malignant)) OR (Benign Neoplasms)) OR (Neoplasms, Benign)) OR (Neoplasm, Benign)) OR (Benign Neoplasm))) OR AB=((((((((((((((((((Neoplasms) OR (Neoplasms)) OR (Tumors)) OR (Neoplasia)) OR (Neoplasias)) OR (Neoplasm)) OR (Tumor)) OR (Cancer)) OR (Cancers)) OR (Malignant Neoplasm)) OR (Malignancy)) OR (Malignancies)) OR (Malignant Neoplasms)) OR (Neoplasm, Malignant)) OR (Neoplasms, Malignant)) OR (Benign Neoplasms)) OR (Neoplasms, Benign)) OR (Neoplasm, Benign)) OR (Benign Neoplasm))

#3 (TI=(((((((((((Anxiety) OR (Anxiety)) OR (Angst)) OR (Nervousness)) OR (Hypervigilance)) OR (Social Anxiety)) OR (Anxieties, Social)) OR (Anxiety, Social)) OR (Social Anxieties)) OR (Anxiousness)) OR (((((((Depression) OR (Depression)) OR (Depressive Symptoms)) OR (Depressive Symptom)) OR (Symptom, Depressive)) OR (Emotional Depression)) OR (Depression, Emotional))) OR ((((((Quality of Life) OR (Quality of Life)) OR (Life Quality)) OR (Health-Related Quality Of Life)) OR (Health Related Quality Of Life)) OR (HRQOL)))) OR AB=(((((((((((Anxiety) OR (Anxiety)) OR (Angst)) OR (Nervousness)) OR (Hypervigilance)) OR (Social Anxiety)) OR (Anxieties, Social)) OR (Anxiety, Social)) OR (Social Anxieties)) OR (Anxiousness)) OR (((((((Depression) OR (Depression)) OR (Depressive Symptoms)) OR (Depressive Symptom)) OR (Symptom, Depressive)) OR (Emotional Depression)) OR (Depression, Emotional))) OR ((((((Quality of Life) OR (Quality of Life)) OR (Life Quality)) OR (Health-Related Quality Of Life)) OR (Health Related Quality Of Life)) OR (HRQOL)))

#4 #1 AND #2 AND #3

CNKI

#1 (Subject: 身心运动) OR (Title/Abstract/Keywords: 身心运动 OR 中医传统功法 OR 六字诀 OR 五禽戏 OR 八段锦 OR 易筋经 OR 瑜伽 OR 气功 OR 太极拳)

#2 (Subject: 肿瘤) OR (Title/Abstract/Keywords: 肿瘤 OR 癌症 OR 恶性肿瘤 OR 良性肿瘤)

#3(Subject: 焦虑) OR (Title/Abstract/Keywords: 焦虑 OR 抑郁 OR 情志

	异常 OR 抑郁症状 OR 抑郁情绪 OR 生活质量 OR quality of life OR life quality OR living quality OR 生存质量 OR 生命质量)<br>#4 #1 AND #2 AND #3
Wanfang Data	#1 (SU = 身心运动) OR (TI/KW = 太极拳 OR 气功 OR 瑜伽 OR 易筋经 OR 八段锦 OR 五禽戏 OR 六字诀 OR 中医传统功法 OR 身心运动)<br>#2 (SU = 肿瘤) OR (TI/KW = 肿瘤 OR 癌症 OR 恶性肿瘤 OR 良性肿瘤)<br>#3 (SU = 焦虑) OR (TI/KW = 焦虑 OR 抑郁 OR 生活质量 OR 情志异常 OR 抑郁情绪 OR 抑郁症状 OR quality of life OR life quality OR living quality OR 生存质量 OR 生命质量)<br>#4 #1 AND #2 AND #3
VIP Database	#1 (M=(身心运动 or 中医传统功法 or 六字诀 or 五禽戏 or 八段锦 or 易筋经 or 瑜伽 or 气功 or 太极拳))<br>#2 (M=(肿瘤 or 癌症 or 恶性肿瘤 or 良性肿瘤))<br>#3 (M=(焦虑 or 抑郁 or 生活质量 or 情志异常 or 抑郁情绪 or 抑郁症状 or quality of life or life quality or living quality or 生存质量 or 生命质量))<br>#4 #1 AND #2 AND #3

Supplementary Table 1. Search strategy

2. Supplementary Table 2

Results	Study removed	Pooled effect size after removing this study, SMD (95% CI)	Heterogeneity (I^2), %
Anxiety	Cramer et al (33)	-0.48 [-0.73, -0.22]	0
	Liu et al (36)	-0.36 [-0.81, 0.08]	42
	Vargas-Román et al (43)	-0.33 [-0.62, -0.05]	16
	Wei et al[a] (44)	-0.36 [-0.75, 0.03]	41
Depression	Chen et al (31)	-0.41 [-0.66, -0.15]	65
	Cohen et al (32)	-0.36 [-0.63, -0.09]	67
	Cramer et al (33)	-0.36 [-0.63, -0.09]	67
	Kiecolt-Glaser et al[b] (35)	-0.37 [-0.67, -0.07]	65
	Liu et al (36)	-0.32 [-0.60, -0.03]	65
	Ratcliff et al (41)	-0.39 [-0.66, -0.12]	63
	Vargas-Román et al (43)	-0.35 [-0.62, -0.08]	67
	Wei et al[a] (44)	-0.29 [-0.55, -0.04]	61
	Yao et al[b] (47)	-0.25 [-0.44, -0.06]	33
	Excluding all SD estimated studies.	-0.26 [-0.51, -0.02]	42
Fatigue	Chandwani et al (29)	-0.30 [-0.71, 0.11]	69
	Chang et al (30)	-0.42 [-0.88, 0.03]	78
	Chen et al (31)	-0.56 [-0.86, -0.26]	46
	Cohen et al (32)	-0.46 [-0.88, -0.04]	77
	Namazinia et al (38)	-0.38 [-0.84, 0.09]	79
	Vadiraja et al (42)	-0.30 [-0.71, 0.11]	72
Sleep quality	Chen et al (31)	-0.80 [-1.35, -0.25]	77
	Ratcliff et al (41)	-0.74 [-1.46, -0.02]	87

	Wen et al[a] (45)	-0.60 [-1.31, 0.10]	87
	Wu et al (46)	-0.38 [-0.86, 0.10]	79
	Yao et al[b] (47)	-0.52 [-1.16, 0.11]	85
HRQOL	Chang et al (30)	0.41 [0.18, 0.64]	61
	Chen et al (31)	0.46 [0.28, 0.64]	35
	Eyigor et al[a] (34)	0.41 [0.19, 0.63]	61
	Liu et al (36)	0.38 [0.13, 0.63]	63
	Molassiotis et al[b] (37)	0.38 [0.13, 0.62]	63
	Namazinia et al (38)	0.41 [0.18, 0.64]	61
	Oh.B. et al,2010[a,b] (39)	0.37 [0.13, 0.61]	62
	Oh.B. et al,2011[b] (40)	0.35 [0.13, 0.58]	60
	Wei et al[a] (44)	0.32 [0.13, 0.52]	46
	Wen et al[a] (45)	0.35 [0.12, 0.58]	60
	Yao et al[b] (47)	0.38 [0.14, 0.62]	63
	Excluding all high risk of bias trials.	0.30 [-0.03, 0.64]	72
	Excluding all SD estimated studies.	0.27 [0.04, 0.50]	49

[a] High risk of bias trial.

[b] SD estimated.

Supplementary Table 2. Summary of sensitivity analyses across outcomes

3. Supplementary Table 3

Outcome indicators	Participants (studies)	Risk of bias	Inconsistency	Indirectness	Imprecision	Publication bias	SMD (95% CI)	Certainty
Anxiety	281 (4 RCTs)	serious[a]	not serious	not serious	serious[b]	none	-0.39 [-0.65, -0.13]	⊕⊕◯◯ Low[a,b]
Depression	765 (9 RCTs)	serious[a]	not serious	not serious	not serious	none	-0.34 [-0.59, -0.1]	⊕⊕⊕◯ Moderate[a]
Fatigue	437 (6 RCTs)	not serious	not serious	not serious	serious[c]	none	-0.4 [-0.78, -0.02]	⊕⊕⊕◯ Moderate[c]
Sleep quality	359 (5 RCTs)	serious[a]	serious[d]	not serious	serious[b]	none	-0.6 [-1.15, -0.05]	⊕◯◯◯ Very low[a,b,d]
HRQOL	879 (11 RCTs)	serious[a]	not serious	not serious	serious[b]	none	0.38 [0.17, 0.6]	⊕⊕◯◯ Low[a,b]

[a] Downgraded for study limitations: one or more included trials were rated as high risk of bias according to ROB2.

[b] Downgraded for imprecision: the confidence interval crossed the line of no effect or statistical significance was lost in sensitivity analyses.

[c] Downgraded for imprecision: the confidence interval crossed the line of no effect.

[d] Downgraded for inconsistency due to substantial heterogeneity.

Supplementary Table 3. Summary of findings and certainty of evidence using GRADE
